# Adult-onset IgE-mediated food allergy at a Winnipeg allergy clinic: a case series

**DOI:** 10.1186/s13223-020-00483-5

**Published:** 2020-09-29

**Authors:** Graham Walter, Chrystyna Kalicinsky

**Affiliations:** 1 Department of Internal Medicine, University of Manitoba, Winnipeg, Canada; 2 Section of Allergy and Clinical Immunology, University of Manitoba, Winnipeg, Canada

**Keywords:** Adult-onset, IgE-mediated, Food allergy, Food-dependent exercise-induced anaphylaxis, Anaphylaxis, Pollen-food syndrome

## Abstract

**Background:**

It is a putatively understood phenomenon that the overall prevalence of allergic disease has been increasing in recent decades—particularly in industrialized nations. Despite this, there is a relative scarcity of data concerning the development of food-related allergic disease in the adult population. In addition, the paucity of data as it pertains to the Canadian population is particularly marked when compared to other nations. We sought to determine common culprit foods and the reactions they elicited in a series of 14 patients seen in the Winnipeg allergy and immunology clinic.

**Methods:**

We conducted a retrospective review of patients identified by academic allergists in Winnipeg, Manitoba as fitting criteria for adult-onset IgE-mediated food allergy from May 2018–July 2020. We included patients with IgE-mediated symptoms, including the pollen-food syndrome which developed at the age of 16 or later. We collected data regarding the food which induced the reaction, what the reaction was, and any concomitant atopic disease.

**Results:**

The most common culprit food identified was shellfish, followed by finfish, pollen-food syndrome, and wheat/flour. The most common reaction experienced was anaphylaxis, followed by food-dependent exercise-induced anaphylaxis and isolated (muco)cutaneous symptoms. With regard to concomitant atopic disease, allergic rhinitis/rhinoconjunctivitis stood out as the most prevalent.

**Conclusions:**

Adult-onset food allergy—particularly with resultant anaphylaxis—is an important phenomenon to recognize, even when patients have previously tolerated the food in question.

## Background

The term “food allergy” is colloquially used to characterize a spectrum of adverse immunologic reactions following exposure to food. When the underlying etiology is demonstrated to be immunoglobulin E (IgE)-mediated, the term “IgE-mediated food allergy” provides a much more specific and accurate description [1]. IgE-mediated food allergies are characterized by their rapidity of onset upon repeat exposure to an allergen following a process of sensitization, with resultant symptoms including urticaria, angioedema (cutaneous and mucocutaneous), pollen-food syndrome (PFS), rhinitis, anaphylaxis, and food-dependent exercise-induced anaphylaxis (FDEIA) [2]. Sensitization can occur following oral ingestion of a food, or through a cross-reactive aeroallergen [3–6]. It is commonly understood that the prevalence of IgE-mediated food allergy has been increasing in recent decades, particularly as it pertains to industrialized countries [7–9].

There are 4 distinct varieties of IgE-mediated food allergy—classic anaphylactic type reactions, PFS/oral allergy syndrome (OAS), delayed anaphylaxis, and FDEIA [10]. It is agreed that individual reactions to certain allergenic foods may persist into adulthood (most commonly peanuts, tree nuts, and seafood), while many reactions disappear with age [10]. Further expansion of this knowledge as it pertains to an adult population is of importance, as food allergy contributes significantly to dietary restriction and decreased quality of life [11]. Aside from these outcomes, anaphylaxis—a systemic and life-threatening disease process which can culminate in hemodynamic collapse and death [12]—is an evidently significant subgroup of IgE-mediated reactions for which current data in our local adult population is also lacking.

The OAS, as coined by Amlot et al. in 1987 [13] is a crucial area of discussion as it relates to symptom burden. This condition is defined as the presence of limited perioral symptoms—throat tightness, lip swelling, and oral mucosal blebs [14]—when exposed to a food (typically a fruit or vegetable). Although rarely seen, reactions as severe as anaphylaxis have occurred from OAS [7]. This phenomenon is believed to be mediated by cross-reactivity between antibodies to an aeroallergen—such as birch, mugwort, or ragweed—and proteins present in foods patients consume [3]. A more descriptive term has been used in recent years to describe this pathophysiology; the pollen-food syndrome [14]. In fact, in 2008 it was proposed that an even more all-encompassing term be used to represent the same processes—food contact hypersensitivity syndrome—to include latex-fruit syndrome which is believed to be on the PFS spectrum [15]. All of these reactions represent an IgE-mediated process which is heavily represented in the adult-onset IgE-mediated allergy population [16].

FDEIA, another principal area of review, is defined by a type I hypersensitivity reaction wherein symptoms are not experienced following exercise or food intake in isolation, but rather after a combination of these culprits [17]. The most common perpetrator of FDEIA is wheat [17, 18], however numerous other causative foods have been identified. Although somewhat similar in pathophysiology to cholinergic urticaria, FDEIA is distinguished by a negative passive heat challenge [19]. In addition to exercise, fatigue, cold, and lack of sleep are all believed to exert some effect on the development of FDEIA symptoms [17, 19].

Accurate epidemiologic tracking of allergy and atopic disease in the adult population has been notoriously difficult. This is particularly problematic with regard to adult-onset IgE-mediated food allergy, as the bulk of contemporary data focuses on the pediatric population [10, 16, 20, 21]. Whether this phenomenon is due to low referral bias regarding less severe allergic disease, or due to a lack of emphasis on tracking of allergic disease owing to the perceived benign nature of many reactions is a matter of debate. To outline the gaps that exist, we look to one such study that attempted to remedy this. This was a survey-based cross-sectional study of food allergy at Imam Abdulrahman Bin Faisal University (IAU), with 5497 students surveyed regarding presence or absence of allergy, and age of onset of food allergy if applicable [22]. This revealed that amongst those with a positive history for food allergy (174 students with clinically diagnosed food allergy of the 526 students who had a positive screening history), 51.7% developed allergy before the age of 14; with adolescent (29.1%) and adult development (19.2%) being less common [22]. This aptly summarizes the relatively large proportion of patients developing food allergy at an adult age, but as with many other studies it fails to outline specific details regarding adult-onset allergy, including reaction and culprit food.

A separate retrospective review of an adult emergency department in Qatar revealed that of 198 patients identified with adult-onset anaphylaxis, 19% of reactions were following food ingestion (background incidence 16.5 cases/100,000 visits), with 29% of the food reactions being to seafood [23]. The applicability of these figures to a Canadian population is quite debateable, further delineating the need for more local data.

One such study by Ruiz et al. attempted to track anaphylactic presentations to a single centre emergency department in Calgary, Alberta in 2011 and 2015. They found that among those presenting with anaphylaxis, the food culprit most often implicated was peanuts (19.7% of food anaphylaxis cases in 2011; 19.7% in 2015), tree nuts (16.7%; 16.9%), and shellfish (12.1%; 8.5%) [24]. The shortcomings of this study as it pertains to adult-onset food allergy are twofold; first, the particular emergency department studied is not described in detail with regard to pediatric and adult patients, and second, there exist no qualifiers for an adult-onset form of food reaction.

Within, we describe a series of 14 patients seen in the allergy clinic in Winnipeg, Manitoba, who were referred for the question of adult-onset allergy to foods previously tolerated.

## Methods

We conducted a case series of patients assessed for concern of adult-onset IgE-mediated food allergy in both of Manitoba’s two academic allergy clinics from May 2018–July 2020. We defined adult-onset IgE-mediated food allergy as a history in keeping with IgE-mediated allergy—as defined by the immediate onset of symptoms consistent with pollen-food syndrome, isolated (muco)cutaneous symptoms, or anaphylaxis including FDEIA [25]—following food exposure at the age of 16 or older. No patients were identified as presenting with delayed-onset anaphylaxis, and as such we did not attempt to comment on the adult development of delayed-onset anaphylaxis.

The diagnosis was confirmed by epicutaneous testing, with a positive test defined as a wheal and flare reaction when a culprit food was applied to the skin via skin prick. Locally, a positive reaction is defined as 3 mm or more of wheal over-and-above the negative saline control skin prick wheal. Where SPT was unavailable or confounded, the diagnosis was instead confirmed by elevated food-specific serum IgE levels (ImmunoCAP Phadia) based on elevation above the upper limit of normal on local laboratory testing in the correct clinical context. For patients with symptoms and culprit foods classic for PFS, the positive SPT result was not a requirement for inclusion, as skin testing to these allergens with commercial extracts is of low sensitivity [25].

We included the PFS and FDEIA as subgroups within our analysis. Patients under the age of 16, or with a history inconsistent with IgE-mediated food allergy (including food intolerance, toxic effects, and immunologic sensitivity without clinical symptomatology), as well as pre-existing food allergy to similar cross-reactive foods were excluded from our case series.

We extracted demographics such as the specific food, index reaction, age at the time of reaction, and comorbid atopic disease; including any skin prick test (SPT) results to aeroallergens. We recorded any results, food-specific serum IgE levels, and all oral food challenges. Atopic disease was as recorded in patient charts during allergy subspecialist consultation, and was recorded as a binary yes/no with regard to a lifelong past medical history of said atopic condition.

## Results

14 patients were included in our analysis. A prospective list of patients meeting the inclusion criteria was kept by physicians at the academic allergy clinic in Winnipeg, Manitoba beginning in July 2018 and was the basis of these 14 patients, with no patient charts identified as not meeting criteria for inclusion. The majority of patients developed their index reactions before the age of 51 (12/14 patients; 86%), while only 2 patients developed IgE-mediated food-allergy at the age of ≥ 51. As well, male patients were slightly more represented than female (8/14 patients; 57%) (Table 1). 5 patients underwent serum-specific IgE testing to confirm their allergy.

**Table 1:** Background characteristics of patients with adult-onset IgE-mediated food allergy

	Number of patients (%) (N = 14)
Age of index reaction (years)	
16–30	5 (36%)
31–40	4 (29%)
41–50	3 (21%)
51–60	1 (7%)
> 60	1 (7%)
Sex	
Female	6 (43%)
Male	8 (57%)

1 patient who was referred following anaphylaxis to finfish (pickerel) underwent oral challenge to an alternate whitefish (Basa), as they demonstrated a negative SPT and serum-specific IgE to this food and wanted to re-introduce finfish into their diet. Unfortunately, there exist no locally available serum specific IgE testing or commercial SPT extracts for pickerel, and thus these investigations were not performed. Typical practice would be to perform SPT to fresh pickerel, but as the patient did not bring the food with her upon follow-up, this testing was deferred as well. The patient was advised to continue pickerel avoidance given their index reaction of anaphylaxis to pickerel alone.

With regard to specific allergens, our population was most represented by adult-onset shellfish allergy (4/14 patients; 29%) followed by finfish, pollen-food syndrome, and wheat allergy at 2 each. In the FDEIA group, the culprit food identified was wheat in 2 cases and shrimp in 1 case. The majority of reactions experienced by these patients were either anaphylaxis (6 patients; 43%), FDEIA and isolated (muco)cutaneous symptoms (3 patients; 21% each) (Table 2). The 2 patients with adult-onset PFS had reactions to foods including zucchini, celery, and mango. Further to this, both patients with PFS had positive skin prick testing to fresh forms of the foods which elicited their oral symptoms—a well described phenomenon [25]—despite this not being a requirement for inclusion in our study.

**Table 2:** Specific food to which patients developed IgE-mediated reaction and type of reaction experienced

	Number of patients (N = 14)
Allergen	
Shellfish	4 (29%)
Finfish	2 (14%)
PFS	2 (14%)
Wheat/flour	2 (14%)
Treenuts	1 (7%)
Soy	1 (7%)
Beer	1 (7%)
Whey	1 (7%)
Reaction	
Anaphylaxis	6 (43%)
FDEIA	3 (21%)
Isolated (Muco)cutaneous symptoms	3 (21%)
PFS	2 (14%)

*PFS* Pollen-food syndrome, *FDEIA* food-dependent exercise-induced anaphylaxis

**Fig. 1:**
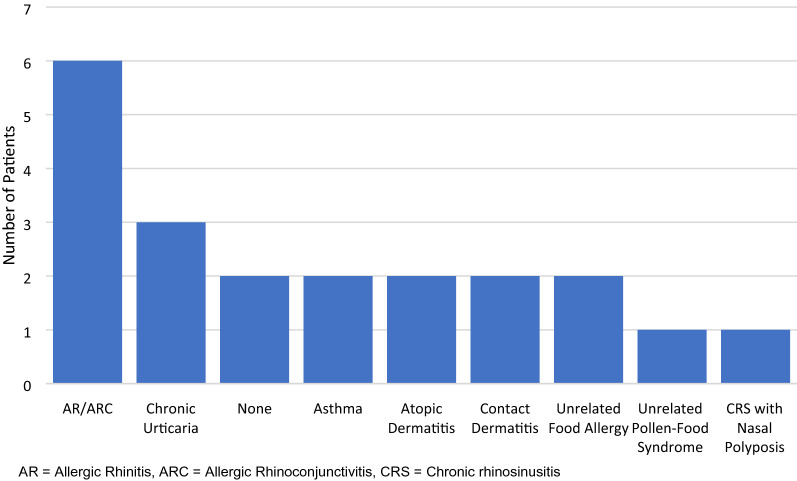
Concomitant allergic disease in patients with adult-onset IgE-mediated food allergy. *AR* Allergic rhinitis, *ARC* Allergic rhinoconjunctivitis, *CRS* Chronic rhinosinusitis

Turning attention to concomitant atopic disease, 6/14 (43%) had a history of allergic rhinitis with or without conjunctivitis, and 3/14 (21%) demonstrated chronic urticaria [1 patient with chronic spontaneous urticaria (CSU), 1 with cold-induced urticaria, and 1 with dermatographism)] (Fig. 1). Of the 6 patients with allergic rhinitis (AR), 4 were positive on SPT to house dust mite (HDM), however only 1 of these patients demonstrated adult-onset shellfish allergy. Of the patients with concomitant AR, 4 of the 7 identified had PFS as their index reaction, while 2 had shrimp (1 positive for HDM, 1 not skin tested for aeroallergens). Only 1 patient reviewed had pre-existing and unrelated PFS, with repeated anaphylaxis to almonds but only isolated oral symptoms following exposure to banana, pear, orange, and cherry.

## Discussion

Our study represents the first local analysis of a significant number of adult-onset IgE-mediated food allergic patients in Manitoba. Findings of this case-series agreed with the retrospective review by Kamdar et al. at Northwestern University in 2015 regarding the most common culprit food being seafood [21]. As was the case with this study, we did not attempt to analyze shellfish subtypes (mollusks vs. crustaceans). With respect to diagnoses, all patients aside from one had positive SPT to the allergens in question. The patient without positive epicutaneous testing underwent food-specific serum IgE levels, which demonstrated significant elevation. No patients included in this case-series underwent oral-food challenge, likely due to the selection bias for inclusion.

In a 2012 Canadian review, the overall prevalence of food “allergy” was 8.07%, with the most common self-reported allergens being shellfish (1.91% of those surveyed), cow’s milk (1.89%), fruits (1.61%), vegetables (1.29%), tree nuts (1.07%), wheat (0.86%), peanuts (0.78%), and hen’s egg (0.67%) [26]. As with the majority of compendia on this pathophysiology, there were no qualifiers given for an adult-age of onset, and the self-reported nature of this data limits its usefulness.

A more objective 2004 cross-sectional study on German patients of all age groups found a 2.6% prevalence of adverse food reactions among a 4093 patient sample population, with confirmatory clinical testing to support this prevalence. Within this study, the most common allergens with confirmatory IgE testing were nuts, apples/pears, stone fruit, vegetables, other fruit, flour, milk, and egg [27].

Again, the issue of which allergens present with adult-onset symptoms fails to be outlined by either of the aforementioned studies. A more recent study in JAMA by Gupta et al. in the US population did include an adult-onset identifier in their patient subgroupings [8]. Of the 40,443 adult who completed their survey, 5.2% (4.9–5.4; 95% confidence interval) of respondents fulfilled criteria for adult-onset food allergy. The study then outlines the most common foods to which patients had allergy—shellfish (2.9%), milk (1.9%), peanut (1.8%), tree nut (1.2%), and finfish (0.9%)—however, once again the reporting of these allergens does not distinguish between adult-onset and pediatric-onset persistent food allergy [8]. Further to this, no Canadian patients were included, limiting the clinical applicability.

Interestingly, of our patients found to be shellfish allergic, only 1/4 had documented epicutaneous testing to house dust mite (HDM) in their medical records, while 2 other patients were not tested for any aeroallergens as they did not have clinical AR, and 1 patient had the documented diagnosis of allergic rhinitis, but results of inhalant allergen testing were not documented in either electronic or physical medical records. HDM-shellfish cross-reactivity is a well-documented phenomenon, believed to be secondary to the high sequence homology between tropomyosin proteins of these organisms [4, 5]. This hypothesis stands analogous to the PFS/OAS, wherein an aeroallergen results in sensitization and subsequent food allergic reactivity, without ingestion of the food necessary to cause this sensitization. The cockroach also possesses a highly homologous tropomyosin protein to HDM, however given the low prevalence of this aeroallergen in Manitoba, it is not commonly tested on our standard aeroallergen panel.

In vitro subcutaneous dust mite therapy studies have shown worsening of mollusk allergy following initiation [28], lending credence to the thought that they possess shared allergenic proteins with the house dust mite. Further to this, Wong et al. demonstrated significant homology between the allergenic epitopes on snail tropomyosin and HDM [4]. Our study did not divide these patients by type of shellfish allergy due to the relatively low prevalence of documented HDM allergic rhinitis. With regard to our 2 non-AR shellfish allergic patients, we hypothesize that they became sensitized to these allergenic compounds via ingestion rather than aeroallergen cross-reactivity. This is of clinical importance as both patients presented with life-threatening anaphylaxis as their index reaction, accounting for half of the shellfish allergic patients reviewed.

When examining our FDEIA population, 1 of the wheat allergic patients had negative skin testing to wheat extract but positive testing to fresh flour. This phenomenon has been demonstrated previously in a small case series of wheat-dependent exercise-induced anaphylaxis [18]. The pathophysiology behind this unique occurrence has yet to be elucidated, but is presumably related to allergen alteration during processing. In similar fashion, the patient who demonstrated true IgE-mediated allergy to whey had negative skin prick testing to commercial dairy extract as well as fresh cow’s milk.

It cannot be emphasized enough how significant the high proportion of anaphylaxis was in this review. The serious nature of this reaction notwithstanding, 39% of food allergic US adults report at least 1 visit to an emergency department for anaphylaxis in their lives, and 9% report at least 1 visit in the last year based on a JAMA review [8]. These staggering statistics are a clear reflection of the cost—both monetary and otherwise—of severe allergic food reactions. A caveat to this point, however is that of referral bias; through which one can conclude that more significant allergic reactions were likely selectively referred to our allergy subspecialty clinic, and more minor reactions were unlikely to have been deemed severe enough to necessitate allergy consultation.

In the aforementioned study by Ruiz et al, the most common culprit foods which resulted in anaphylaxis that necessitated emergency department presentation were peanuts followed by tree nuts and shellfish [24]. In our studied patients, however, culprit foods for non-exercise-dependent anaphylaxis varied, with a shellfish culprit in 2/6 patients, and soy, finfish, whey, and almonds culprits in 1 patient each. The differences between their study and ours can be explained by the low number of patients included in our review, the possible inclusion of pediatric patients and the lack of qualifiers regarding FDEIA in the Ruiz study, and by our specification that patients included have an adult-onset reaction to foods previously tolerated.

Unfortunately, data regarding whether our adult-onset PFS patients immigrated to Canada from a country with differing aeroallergens was not recorded. This is of interest, as it has been demonstrated that in-movers to a new environment demonstrate lower rates of atopic disease, with gradual increases as they become sensitized [29]. This could theoretically provide an explanation as to adult development of cross-reactive allergic disease, as accounted for by the time to sensitization. The prevalence of PFS overall (1 pre-existing, 2 adult-onset) was fairly consistent with results published by Ma et al. who reported an estimated prevalence of PFS of 8% in the general population based upon a sample of 250 US allergists [30]. Confounding this finding would be our small sample size and perhaps low referral rates for patients with PFS; although this has not been studied in the Canadian population. Both aforementioned adult-onset PFS patients had a history consistent with allergic rhinitis, in keeping with the cross-reactivity hypothesis of PFS [3].

The proportion of concomitant allergic rhinitis was expectedly high given the number of patients with PFS and adult-onset shrimp allergy. This was expected based upon the stated pathophysiology of shrimp allergy and PFS. One almond allergic patient also demonstrated AR with positive SPT to trees (a common cross-reactor with almonds), however their index reaction was anaphylaxis, and thus it was concluded that this was not in keeping with PFS, given the relative rarity of anaphylaxis with pure PFS, and the lack of (peri)oral tingling accompanying their reaction [7]. We also felt it was important to distinguish this reaction from classically described PFS given its life-threatening nature.

Although most adult-onset allergy studies to date use the accepted legal definition of “adult” as aged 18 and older [10, 16, 21, 23, 31], we defined this with an age of 16 years or older as our cut-off. Although this resulted in only 1 additional case in our series, we believe this is a valid exclusion point, as the age 16 and older has been used in oral immunotherapy (OIT) Canadian guidelines [32]. This is reflective of the loss of immune plasticity following childhood, which has been as a reduction in OIT response by 17% for each year after the age of 5 [33]. It is also local practice that patients aged 16 or older are often referred for assessment by an adult allergist rather than a pediatric allergist in an academic setting.

Our conclusions are limited by the retrospective nature of this review and the fact that not all patients underwent the same diagnostic tests (i.e., not all patients were skin tested for aeroallergens). Further to this, the small number of cases included in this review may have skewed our data, and further study and extension of this data would be essential to expand our understanding of the true prevalence of this pathology.

## Conclusion

Our case series demonstrates that a significant number of patients referred to our local allergy clinic for adult-onset food allergy presented with anaphylaxis and FDEIA as their index reaction. Shellfish was the most common culprit, with all patients having tolerated the food prior to their index reaction. This is significant, as it represents a commonly ingested food item in North America, and a significant reaction with outcomes including morbidity and mortality.

## Data Availability

The datasets generated and/or analysed during the current study are not publicly available due to individual health data privacy, but are available from the corresponding author upon reasonable request.

## Abbreviations

AR: Allergic rhinitis
ARC: Allergic rhinoconjunctivitis
CRS: Chronic rhinosinusitis
CSU: Chronic spontaneous urticaria
FDEIA: Food-dependent exercise-induced anaphylaxis
PFS: Pollen-food syndrome
HDM: House dust mite
IgE: Immunoglobulin E
OAS: Oral Allergy Syndrome
OIT: Oral immunotherapy
SPT: Skin prick testing
